# Macrophage activation syndrome during treatment with biological therapy in patients with systemic juvenile idiopathic arthritis

**DOI:** 10.1186/1546-0096-12-S1-P214

**Published:** 2014-09-17

**Authors:** Alina Lucica Boteanu, Maria Angeles Blazquez Cañamero, Valentina Maldonado Romero, Maria Luz Gámir Gámir

**Affiliations:** 1 Rheumatology, University Hospital Ramon y Cajal, Madrid, Spain

## Introduction

Macrophage activation syndrome (MAS) is a severe complication of autoimmune diseases. It is more often associated with Systemic Juvenile Idiopathic Arthritis (sJIA). It is difficult to distinguish the MAS from a flare of sJIA, infection or medicinal side effects. The MAS can occur during a flare, an infection, a change in the medication or as a side effect of the treatment administered for the treatment of sJIA. Paradoxically have been reported MAS cases in patients with sJIA treated with anti IL-1 or anti IL-6R.

## Objectives

We report two cases of sJIA who developed MAS during the treatment with biological therapy.

## Methods

Revison of two cases of MAS associated with sJIA treated with biological therapy in our clinic.

## Results

### Case1

Girl diagnosed at 9 years of sJIA, treated with Prednisone and then MTX for persistent arthritis. A few yers later, Anakinra treatment is started for persistent joint activity despite MTX, suspended after 5 weeks of treatment for suspected unconfirmed septic arthritis, treated with antibiotics and anti-inflammatory. A month after the resolution of the infectious process it was restart Anakinra, detecting eight days later deterioration of liver function and subsequent development of a analytical and clinically symptoms compatible with MAS. The clinical picture improved gradually after initiation of cyclosporine and high dose of prednisone, achieving complete remission. Currently treated with Tocilizumab with good clinical outcome, without new MAS episode.

### Case2

Patient diagnosed of sJIA according to ILAR criteria at 2 years of age, debuting with tipically clinical, laboratory and histological MAS, coinciding with very high disease activity . At 4 years of age it is initiated Etanercept that is suspended 1 year later for ineffectiveness. At age 8 he start Anakinra with good clinical response, treatment that is maintained up to 14 years when it is suspended for clinical remission and local side effects (pain). Six months after the suspension he presents a new flare with new joint and systemic activity that is treated with Tocilizumab alone, without Prednisone. After the second infusion of Tocilizumab he presents upper respiratory infection, treated with Amoxicillin and anti-inflammatory treatment. Several days later he is hospitalized with clinical and laboratory tests compatible with MAS. The bone marrow biopsy confirmed the presence of hemofagocitos. The microbiological tests detected recent Ebstein Barr Virus infection. It was initiated treatment with Cyclosporine i.v Methylprednisolone with resolution of symptoms.

## Conclusion

To our knowledge, there are reported a few cases of MAS coinciding with the anti IL -1 or anti IL -6R treatments administered sJIA therapy. In recently published clinical trials, conducted to evaluate the efficacy and safety of treatment of IL -1beta monoclonal antibody and anti IL-6 receptor antibody, it was detected a few cases of MAS. The mechanism of occurrence of MAS during these therapies is still unclear. The hypothesis that arises is that the blockade of proinflammatory cytokines and /or an infectious trigger could create an imbalance in the regulation of cytokines and activation of T lymphocytes and macrophages with a significant increase of proinflammatory cytokines, which may trigger a macrophage activation syndrome. Our two patients have had prior to the development of MAS an intercurrent infection, receiving antibiotic and anti-inflammatory treatment. The second case had a history of SAM prior to any treatment.

## Disclosure of interest

None declared.

